# Measurement of Typhim Vi IgG as a Diagnostic Tool to Determine Anti-polysaccharide Antibody Production Deficiency in Children

**DOI:** 10.3389/fimmu.2019.00654

**Published:** 2019-04-02

**Authors:** Kissy Guevara-Hoyer, Celia Gil, Antony R. Parker, Leigh J. Williams, Carmen Orte, Antonia Rodriguez de la Peña, Juliana Ochoa-Grullón, Edgard Rodriguez De Frias, Irene Serrano García, Sonia García-Gómez, M. José Recio, Miguel Fernández-Arquero, Rebeca Pérez de Diego, Jose Tomas Ramos, Silvia Sánchez-Ramón

**Affiliations:** ^1^Department of Immunology, IML and IdSSC, Hospital Clínico San Carlos, Madrid, Spain; ^2^Department of Immunology, Ophthalmology and ENT, School of Medicine, Complutense University, Madrid, Spain; ^3^Immunodeficiency Interdepartmental Group (GIID), Madrid, Spain; ^4^Department of Pediatrics, Hospital Clínico San Carlos, Madrid, Spain; ^5^The Binding Site Group Limited, Birmingham, United Kingdom; ^6^Department of Epidemiology and Preventive Medicine, Hospital Clínico San Carlos, Madrid, Spain; ^7^Laboratory of Immunogenetics of Human Diseases, IdiPAZ Institute for Health Research, Madrid, Spain

**Keywords:** Typhim Vi, children, polyssaccharide, vaccine, antibody deficiencies, immunodefiency

## Abstract

**Background:** The assessment of specific polysaccharide antibody production plays a pivotal role in the diagnosis of humoral primary immunodeficiencies (PID). The response to 23-valent pneumococcal vaccine (PPV) remains the gold standard for the diagnosis of polysaccharide antibodies. However, in Spain, the interpretation of pure polysaccharide 23-valent immunization is hampered by the high endemicity of pneumococcal disease and the generalization of the 13-valent adjuvant pneumococcal vaccination. Specific Typhim Vi vaccination (TV) immunoglobulin G IgG response to immunization is useful in adult PID, but there is no data regarding children.

**Objectives:** To evaluate the clinical utility of TV IgG production as a diagnostic tool to determine anti-polysaccharide antibody production deficiency in children, when the response to PPV is unclear and isolated determination of serotypes is unfeasible.

**Methods:** We conducted a single-institution prospective observational study on 61 children with recurrent infections. Baseline specific antibodies against PPV and TV were evaluated. In 28 children (46%), the response to the production of antibodies confirmed a clinical suspicion of humoral PID, and they were therefore immunized with 23-valent pneumococcal vaccine and Typhim Vi. Both specific antibody responses were measured by ELISA (The Binding Site Group Ltd, Birmingham, UK) using previously published cut-offs.

**Results:** Seventy percent of the 61 children displayed baseline PPV IgG > 27 mg/L, whereas only 8% showed TV IgG > 28 U/mL (*p* < 0.0001). Twenty-one of 28 children (75%) achieved a 3-fold increase in post-vaccination TV IgG levels, whereas only 3% achieved a 4-fold increase in PPV IgG post vaccination, mainly due to high baseline PPV IgG titers. When we classified children according to their response to TV as responders or non-responders and compared this with the well-known clinical warning signs of the Jeffrey Modell Foundation. The proportions of children with history of pneumonia and the need for intravenous antibiotics were significantly higher in TV IgG non-responders than in TV IgG responders (*p* = 0.02 and *p* = 0.01, respectively).

**Conclusion:** Response to TV can be considered an ancillary diagnostic tool to determine polysaccharide antibodies in children, particularly when isolated determination of pneumococcal serotypes is not feasible. TV provides a useful asset for clinicians in the era of conjugate PPV vaccination, with clinical relevance. Further research is warranted for validation.

## Introduction

### Current Challenges Posed by the Pneumococcal Vaccine in the Evaluation of Primary Immunodeficiency in Children

The inability to produce an adequate and protective immune response to polysaccharides antigens present on the surface of pathogens renders individuals susceptible to recurrent and severe sinopulmonary infections due to encapsulated bacteria. In patients with suspected primary immunodeficiencies (PID), an early and accurate diagnosis of antibody production deficiency will provide the individual with prompt and more appropriate treatment (1, 2).

The current gold-standard test to evaluate the response to the polysaccharide vaccine is based on the measurement and interpretation of the antibody response to the 23-valent pneumococcal polysaccharide vaccine (PPV) (3). However, the undoubted success of the pneumococcal conjugate vaccine, which is included in the Spanish childhood vaccination schedules and in the at-risk group patients, as recommended by the CDC, has made interpretation more complicated (1, 4, 5). By increasing the difficulty of PPV interpretation, the definition of a standard response may be influenced by herd immunity following generalized immunization in childhood and the endemic exposure to pneumococcus (6, 7).

A meta-analysis of anti-pneumococcal antibody responses on an individual serotype basis in healthy individuals showed that the ratios of pre- and post-vaccination titters varied widely and depended on the particular antibody serotype and baseline levels [4]. There is not a clear consensus from laboratories between the measurement and importance of each individual serotype response and the total value of the pneumococcal polysaccharide IgG vaccine (3, 8–10). Likewise, the assessment of each specific serotype requires a single ELISA, which is time-consuming and expensive to be applied in the routine practice and is therefore available at only a few highly qualified laboratories in Spain for research purposes (8).

Borgers et al. (21) analyzed the vaccine responses to Pneumovax® in healthy children and adults and in patients referred with recurrent infections and/or a diagnosis of specific antibody deficiency, showing good correlation between total pneumococcal IgG levels in PPV and the number of serotypes to which they responded (6).

On the other hand, it is assumed that a healthy immune response to polysaccharide vaccine normally develops by 2 years of age, but this is extremely variable and may take longer in some children (11, 12).

Therefore, the interpretation of this vaccine response comes with some challenges and can be complex (3). Alternative new antigen immunization to determine polysaccharide vaccines responses, such as Typhim Vi (TV) vaccines, is now being considered for diagnostic use. The response to TV vaccines has been reported in healthy populations and documented in patients with both PID and secondary immunodeficiency (SID) (1, 2, 13–17). Furthermore, the use of a combination of two different polysaccharide vaccines to assess antibody deficiency may be of interest.

TV is a capsular polysaccharide vaccine, administrated to populations at risk of developing typhoid fever or to individuals traveling to endemic areas, licensed in 1988 for assessment of adaptive immunity in adults and children > 18 months of age (4, 13). Given the lack of a reliable anti-typhi Vi IgG standardization, several studies have measured the pre- and post-vaccination concentration ratio, demonstrating a median 3-fold increase in more than 95% of the cohorts (14, 15). Similarly, the utility of evaluation for humoral immune response patients with PID was shown in recent studies (2, 3, 8, 10, 13).

At present, there is very little evidence supporting TV vaccination for the assessment of adaptive immunity in a pediatric population (14). It has been shown that baseline concentrations of TV IgG are low in children (14, 15). There are no reports of the utility of TV in children that have received pneumococcal conjugate vaccine.

The main purpose of the current study was to explore the potential value of TV vs. PPV as a diagnostic tool to analyze the response to a pure polysaccharide vaccine in a pediatric population that has received both pneumococcal conjugated vaccine and 23-valent pneumococcal vaccine.

## Methods

### Subjects

This was a single-institution, observational study, conducted at the Hospital Clínico San Carlos, Madrid, Spain.

Sixty-one children with recurrent upper and/or lower respiratory tract infection (*n* = 61) were referred to the Pediatric Department. Pre-vaccination concentrations of pneumococcal and *Salmonella Typhi* IgG antibodies were determined. In 28 of the 61 children, natural antibody titters raised the clinical suspicion of humoral primary immunodeficiency, and they were therefore immunized with PPV (Merck) and TV (Sanofi Pasteur).

After a median interval of 35 days, post vaccinations, blood was drawn from all 28 subjects. Pre- and post-immunization serum samples were separated by centrifugation and then stored in aliquots at −40°C for analysis. Twenty-three out of 28 patients had received pneumococcal conjugate vaccine as part of their childhood vaccination schedule. Therefore the PPV pre-vaccination concentration represents the maintenance response to pneumococcal conjugate vaccine. None of the patients had previously received intravenous immunoglobulin therapy or immunosuppressive treatment. As this is an observational study, describing only the results of a routine intervention at our center to measure antibody production, no informed consent was required. The study was approved by the center's ethics committee.

### Assays

Specific antibodies raised against TV and PPV were measured using commercial ELISA kits; VaccZyme™ human anti-*Salmonella* Typhi Vi IgG TV IgG and anti-pneumococcal capsular polysaccharide IgG (The Binding Site Group Ltd, Birmingham, UK). Samples were tested according to the manufacturer's instructions.

The following cut-offs were used: pre TV vaccination IgG 28 U/mL (upper normal limit for pre-vaccination TV IgG concentration) (1, 14) and 27 mg/L as the approximate highest concentration of pneumococcal conjugated antibodies obtained in the childhood vaccination schedule in a healthy pediatric population (1, 18). Fold increase in concentration (FI) was determined using the following formula (post-vaccination/pre-vaccination concentration). Responders were found to achieve a FI of 3 for TV IgG and 4 for PPV IgG, respectively (13–15).

### Statistical Analysis

Data were analyzed by Chi-squared, Fisher's exact, Pearson, and Spearman correlation coefficient and Mann Whitney *U*-tests using SPSS (Chicago, Illinois) and GraphPad Prism software (GraphPad Software, La Jolla, CA, USA). A *p*-value of below 0.05 considered as statistically significant was determined by the Student's “*t*” test.

## Results

### Baseline Seroprevalence of Pneumococcal and *Salmonella Typhi* IgG Antibodies in Children

We measured pre-vaccination concentrations for PPV and TV Vi IgG antibodies in the 61 subjects. The median age was 4 years (range, 2–17), with an M:F ratio of 0.6. The median baseline antibody concentrations were 9.8 mg/L for PPV IgG (range 0.33–27.0 mg/L) and <7.4 U/mL for TV IgG (range 7.4–32.7 U/mL), respectively. There was no influence of age on pre-vaccination titers.

A significantly higher percentage of individuals (70%, 43/61) showed pre-vaccination PPV IgG titers above 27 mg/L compared to 8% (5/61) that showed pre-vaccination TV IgG titers above 28 U/mL (*p* < 0.0001), possibly related to previous immunization and exposition.

Pre-TV titers were below 7.4 U/mL in 69% (42/61) of subjects and the remaining 31% (19/61) presented titers below 28 U/mL. Thus, 92% (56/61) of individuals showed no serological evidence of previous exposure to the antigen, meaning that the Typhim Vi antigen might be considered as a neoantigen, as is the case in the vast majority of the adult population in Spain.

### Post Pneumococcal and *Salmonella typhim* IgG antibodies

In 28 of the 61 children, response to antibody production confirmed the clinical suspicion of immunodeficiency. They were therefore immunized with PPV and TV. Post-vaccination TV IgG titers was 55 U/mL (range 7.4–465.0 U/mL). Seventy-five percent (21/28) achieved a 3-fold increase for TV IgG post-vaccination. The patients were categorized into two groups based on their response to TV, pediatric responders (R, *n* = 21), and non-responders (NR, *n* = 7).

Post-vaccination PPV IgG titers were 27 mg/L (range 0.86–27.0 mg/L), although it must be taken into account that the highest total measurement for this antibody are 27 mg/L, meaning that FI would be significantly lower in patients with high pre-vaccination titers (described below).

**Figure 1:**
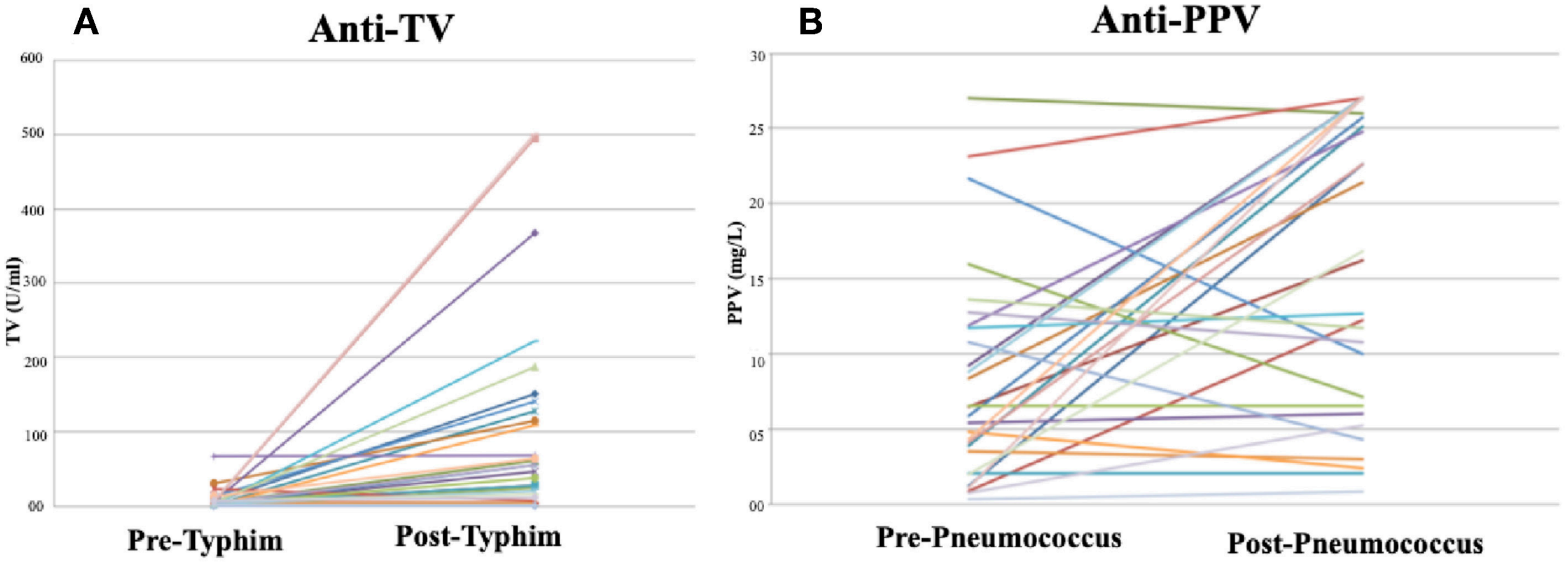
Antibody concentrations Pre- Post TV IgG and PPV IgG in children referred for immunological investigation. **(A)** Pre- Post TV responses, **(B)** Pre- Post PPV responses.

The trend in PPV response between pre- and post-vaccination in the same 28 patients was not the same as that obtained with TV (Figures 1A,B).

Median post-TV IgG concentrations were significantly higher in R (184 U/mL, range 21.4–465.1 U/mL) compared to NR (7.4 U/mL, range 7.4–16.2U/mL, *p* = 0.01). Consistent with this, the median FI was significantly lower in NR (1x; *p* = 0.001) compared to R (12x). The FI in TV and PPV responses were directly compared per patient for all 28 patients. Per patient, the FI for PPV was significantly lower than that obtained for TV (range 0.46–24.55 vs. 0.31–92.92, respectively, *p* = 0.02).

### Clinical Relevance of Responses to TV in Children

We sought to determine the clinical significance of obtaining a TV response in the pediatric population by comparing the clinical characteristics in the R vs. NR when considered the well-known warning signs of PID proposed by the Jeffrey Modell Foundation (Table 1). The percentage of children with recurrent infections and >2 deep-seated infections (such as septicemia or meningitis) were higher in NR with respect to R (NR = 42% vs. *R* = 19%, *p* = 0.31 and NR = 29% vs. *R* = 5%, *p* = 0.14). In addition, the numbers of pneumonia episodes per year and the percentage of patients needing intravenous antibiotics to clear infections was significantly higher (NR = 57% vs. *R* = 19%, *p* = 0.02; and NR = 85% vs. *R* = 3%, *p* = 0.01, respectively).

**Table 1:** Comparison of the TV R and NR clinical characteristics with the Jeffery Modell Warning Criteria for pediatric PIDs.

**Warning signs of a pediatric PID (The Jeffrey Modell Foundation)**	**Responders *n* = 21**	**Non-Responders *n* = 7**	*P*-value^*^
>4 ear infections in 1 year	4 (19%)	3 (42.8%)	0.31
>2 severe sinus infections in 1 year	0 (0%)	0 (0%)	–
>2 months treatment with antibiotic giving Little effect	0 (0%)	0 (0%)	–
>2 pneumonias per year	4 (19%)	4 (57%)	0.02
Insufficient weight gain or growth delay	NA	NA	–
Recurrent deep skin or organ abscesses (including liver and lungs)	0 (0%)	0 (0%)	–
Persistent thrush in mouth or fungal infections on the skin	0 (0%)	0 (0%)	–
Need for intravenous antibiotics to clear infections	3 (3%)	6 (85%)	0.01
>2 Deep seated infections (including septicemia and meningitis)	1 (5%)	2 (28.5%)	0.14
Family history of a PID	NA	NA	-

* *Exact Fisher Test. NA, data not available*.

*Data obtained from http://downloads.info4pi.org/pdfs/10-Warning-Signs—Illustrated*.

Only 4 of 28 children showed isolated lacked response to PPV (FI ≤ 1). Of these 4 patients, only 1 required hospitalization because of severe pneumococcal pneumonia despite being vaccinated with PPV and normal basic analysis and immunological tests. The remaining 3 patients presented with mild symptoms, such as self-limited viral infections. These 4 patients were between 2 and 3 years of age and all of them had previously received the pneumococcal conjugate vaccine as part of their vaccination schedule. Remarkably, all 4 patients showed an adequate response (FI > 3) to TV.

Three out of 7 TV NR while adequately responded to PPV. However, these 3 patients suffered from recurrent low-tract respiratory infections (i.e., pneumonia and bronchiectasis) and associated concurrent immunological alteration (i.e., IgM deficiency, IgA deficiency, and Familial Cold Autoinflammatory syndrome). The remaining 4 patients lacked responses to both PPV and TV, presented recurrent respiratory and gastrointestinal manifestations and intracranial hypertension and were diagnosed with Behçet's disease, partial IgA deficiency, primary antibody deficiency, and common variable immunodeficiency (CVID) (details of the 7 TV NR are shown in Table 2).

**Table 2:** Clinical and serological profiles of TV NR.

**Patients**	**#1**	**#2**	**#3**	**#4**	**#5**	**#6**	**#7**
Gender	F	F	F	F	M	M	M
Age	3	16	6	4	10	9	14
Diagnosis/follow up	IgM deficiency	Bechet's disease	Primary antibody deficiency	Partial IgA deficiency	Common Variable Immunodeficieny	Selective IgA deficiency	Familial Cold Autoinflamamatory síndrome.
Notable clinical features	Recurrent pneumonias CT scan: atelectasis and bronchiectasis	Neutropenia, recurrent respiratory and urinary tract infection, vaginal candidiasis infection	Recurrent bronchitis, bronchiectasis, laryngomalacia, bronchomalacia.	Recurrent respiratory infections, atopic dermatitis and moderate bronchial asthma	Chronic diarrhea, recurrent ear infections. Intracranial hypertension.	Recurrent higher and lower respiratory tract infections (Sinusitis, ear infections, and pneumonia). Bronchiectasis.	Recurrent episodes of fever and systemic inflammation cold-related, higher, and lower respiratory tract infections (Sinusitis, pharyngitis, ear infections, and pneumonia), recurrent bronchiolitis. Bronchiectasis.
TV FI	1	1	0.3	1	1	2.1	1.7
PPV FI	14	0.9	1.2	0.4	2.6	8.4	7
IgG^*^ (g/L)	6.66	10.8227	10.18	11.44	3.10	16.20	7.87
IgG1^*^ (g/L)	4.76	4.34	6.53	7.43	0.34	7.75	4.65
IgG2^*^(g/L)	0.96	4.74	1.59	2.49	0.03	5.63	2.27
IgA^*^(g/L)	0.54	2.12	0.93	0.009	<0.002	<0.002	1.06
IgM^*^(g/L)	0.004	1.84	1.23	1.72	<0.09	0.07	1.1

* *(NV. Parameters were established according to age range)*.

*NA, data not available*.

*All diagnoses were made according the ESID diagnostic criteria*.

No significant differences were found when stratifying patients according to their ages, 2 patients between 2 and 5 years of age, 3 between 5 and 10 and 2 over 10.

## Discussion

High circulating concentrations of pneumococcal antibodies can be a complicating factor when interpreting the PPV response (19). TV pre-vaccination IgG levels have been reported to be low in adults (13, 14) with the majority of individuals having a concentration of <100 U/mL and were lower in children compared to adults (14). The baseline concentrations reported in this study match with previous publications (2, 13, 14). On the other hand, baseline antibody levels against TV were mostly undetectable in the studied population, which, together with the low prevalence against *Salmonella typhi* in Spain, could be considered as a useful neoantigen in the determination of polysaccharide antibodies.

Cautions joint interpretation of baseline antibody titers together with fold-increase is essential to adequately discriminate humoral defects and this interpretation show clinical implications. In our study, we observed that baseline levels of PPV were high at pre-vaccination in up to one-third of patients tested, which could be a reflection of the antibodies generated in previous vaccination against pneumococcal conjugate vaccine. When pre-vaccination titers are already high, immune capacity may be normal despite low “fold-increase” (2, 8, 20). Therefore, a 4-fold increase post vaccination in these children is not to be expected. This could be a confounding factor in determining the production of polysaccharide antibodies if only the gold standard is used, as these individuals did not generate the expected response increase (FI ≥ 4) despite being immunocompetent, which makes it difficult to interpret. While it is true that measuring isolated serotypes against pneumococcus is a useful tool in this regard, in particular in patients with total IgG PPV response >40 mg/L (20), at present no center in Spain actually perform this for routine purposes.

The Spanish childhood vaccination schedule since 2006 recommends the compulsory vaccination of children with a protein-conjugated polysaccharide vaccine as part of a four-stage program (2, 4, 6, and 12–15 months). Measurement of IgG PPV in antibody response may be complicated by prior vaccination with this vaccine (9). We hypothesize that the TV vaccines are failing to mount a primary polysaccharide response whereas the PPV responses could be generated due to a boosted T-dependent pneumococcal conjugate vaccine response. It is, therefore, a current and common problem for the clinician facing PID patients. The strong point of the present study is that the TV response as a pure polysaccharide is a useful complementary diagnostic tool in children whose antibody response to pneumococcal vaccination is difficult to construe.

The main conclusion of our preliminary investigation is that TV seems to be a more reliable indicator of PID/SID. However, in our series of children from the 4 isolated non-responders to PPV that appear to have the worst clinical course, 3 had a self-limiting viral infection and just one confirmed exposure to pneumococcal pneumonia, with no other alterations in the immunological profile. As a result, we hypothesize that this finding could be related to a specific alterations in the T-dependent potentiated functional response associated with immunological immaturity, relying on the fact that the polysaccharide response to TV was adequate. This may be due to the fact that all 4 were 2–3 years-old and previously vaccinated with conjugate pneumococcal vaccine. However, we consider that a medical follow-up of these cases would be recommended, as well as another test to determine the production of antibodies against total and specific PPV for each serotype.

Five out of 28 patients had not received pneumococcal conjugate vaccine as part of their childhood vaccination, since they were born before 2006 or between 2012 and 2015 when this vaccine was not mandatory in the Spanish vaccination calendar. However, when determining the production of antibodies against PPV, this was adequate.

For patients who presented a deficit of production of TV antibodies, a relationship between them was established with other immunological failures, as well as more severe clinical manifestations, showing more consistency for PID diagnosis.

Several studies have reported a variable TV response in healthy volunteers. The FI concentration in healthy children (range, 5–15 years) was between 3- and 8-fold, which may vary according to previous vaccination (3–5). Using this 3-fold cut-off and that reported by others (1, 9), we were able to distinguish between TV R and NR. An inadequate response to TV was associated with a higher percentage of patients with ear infections and more severe infectious diseases such as pneumonia and deep-seated infections. A high percent of NR required more aggressive therapeutic management with the need for hospitalization for administration of intravenous antibiotics compared to TV R. The unclear relationship between the response to TV and the derivative of PPV could be due to the fact that many children had high baseline levels for *Pneumococcus*, possibly derived from previous exposure and from the vaccination schedule.

In our study, we report the measurement of TV IgG as a marker of adaptive immunity in children and illustrate the advantage of measuring the TV response associated with the gold standard compared to measuring and interpreting the isolated PPV response. We confirm that baseline concentrations of TV IgG were low in children compatible with data in adults (1) and that the response to TV can still be used to determine specific polysaccharide antibody production this population with prior pneumococcal conjugate vaccination, even though the interpretation of the gold-standard is more complex in those cases and we show the clinical relevance of measuring the response to TV.

This investigation emphasizes the clinical value of measuring TV IgG as a diagnostic tool for assessing adaptive immunity and aiding the patients' therapeutic decision process.

One limitation in this study is its inability to distinguish between high responses of 23-valent pneumococcal polysaccharide vaccine to a few individual serotypes and a good overall response. Consistently, the impossibility of having healthy control of children, since TV is outside the vaccination schedule, is another limitation and therefore our ethics committee would not have approved it. However, children with benign infections with normal TV response from our cohort may be considered as a control group. Vaccination of healthy children against TV is not required as part of the routine immunization schedule, which led us to extrapolate reference ranges from healthy pediatric populations from previous reports (1, 14).

Moreover, based on published studies, assessment of the TV response could be considered as the additional gold-standard test for assessing polysaccharide production (1, 2, 13, 14, 16). Clearly, there are different implications arising from the response to PPV and given that the polysaccharide structures in both vaccines are different, studies are underway to understand the clinical value of assessing and interpreting both responses together to increase our knowledge of polysaccharide antibody production (1, 10).

## Conclusions

In our cohort, baseline TV antibodies were undetectable in the vast majority of patients and the range of response was wide, reinforcing their utility in clinical practice (2, 10, 13, 16). Although, the sample size was small, the results matched previous studies (15, 16) and in particular those obtained in a pediatric population (1, 14).

We hypothesize that measurement of polysaccharide antibody response using PPV may be influenced by prior exposure of the pneumococcal conjugate vaccine, generating an enhanced T-dependent response. Our current study supports the concept that TV is a useful diagnostic tool for assessing polysaccharide antibodies responses, when the isolated determination of serotypes against pneumococcus is unfeasible, since TV response is not influenced by previous exposure due to the low prevalence of the disease, showing clearer behavior. It supports previous reports detailing the low prevalence of *Salmonella typhi* IgG in the general Spanish population and its role as a neoantigen.

To conclude, we propose that interpretation of the IgG response to TV may be superior to that found for total PPV alone, aimed at evaluating the response to the polysaccharide vaccine in the pediatric population previously vaccinated with the conjugate pneumococcal vaccine.

## Data Availability

The datasets generated for this study are available on request to the corresponding author.

## Author Contributions

KG-H, SS-R, CG, JR, and MF-A contributed conception and design of the study. RP, SG-G, and MR have contributed to the immunological study of PID. CO and AR organized the database. IG performed the statistical analysis. KG-H wrote the first draft of the manuscript. SS-R, ER, JO-G, AP, and LW wrote sections of the manuscript. All authors contributed to manuscript revision, read and approved the submitted version.

### Conflict of Interest Statement

AP and LW are employed by The Binding Site Group Ltd. The remaining authors declare that the research was conducted in the absence of any commercial or financial relationships that could be construed as a potential conflict of interest.
